# Itraconazole treatment of primary malignant melanoma of the vagina evaluated using positron emission tomography and tissue cDNA microarray: a case report

**DOI:** 10.1186/s12885-018-4520-5

**Published:** 2018-06-04

**Authors:** Kayo Inoue, Hiroshi Tsubamoto, Roze Isono-Nakata, Kazuko Sakata, Nami Nakagomi

**Affiliations:** 1 Department of Obstetrics and Gynecology, Hyogo College of Medicine, 1-1 Mukogawa, Nishinomiya, Hyogo 663-8501 Japan; 2 Department of Medical Oncology, Meiwa Hospital, Nishinomiya, Hyogo 663-8186 Japan; 3 Department of Surgical Pathology, Hyogo College of Medicine, 1-1 Mukogawa, Nishinomiya, Hyogo 663-8501 Japan

**Keywords:** Melanoma, Vaginal neoplasm, Itraconazole, Repurposing, Off-use

## Abstract

**Background:**

Primary malignant melanoma of the vagina is extremely rare, with a poorer prognosis than cutaneous malignant melanoma. Previous studies have explored the repurposing of itraconazole, a common oral anti-fungal agent, for the treatment of various cancers. Here, we describe a patient with metastatic, unresectable vaginal malignant melanoma treated with 200 mg oral itraconazole twice a day in a clinical window-of-opportunity trial.

**Case presentation:**

A 64-year-old Japanese woman with vaginal and inguinal tumours was referred to our institution. On the basis of an initial diagnosis of vaginal cancer metastatic to the inguinal lymph nodes, we treated her with itraconazole in a clinical trial until the biopsy and imaging study results were obtained. During this period, biopsies were performed three times, and ^18^F-fluoro-deoxyglucose positron emission tomography (FDG/PET)–computed tomography (CT) was performed twice. Biopsy results confirmed the diagnosis of primary malignant melanoma of the vagina. Imaging studies revealed metastases to multiple sites, including the brain, for which she underwent gamma-knife radiosurgery. During the window period before nivolumab initiation, the patient received itraconazole for 30 days. Within a week of itraconazole initiation, pain in the inguinal nodes was ameliorated. PET–CT on days 6 and 30 showed a reduction in tumour size and FDG uptake, respectively. The biopsied specimens obtained on days 1, 13, and 30 were subjected to cDNA microarray analysis, which revealed a 100-fold downregulation in the transcription of four genes: *STATH*, *EEF1A2*, *TTR*, and *CDH2*. After 12 weeks of nivolumab administration, she developed progressive disease and grade 3 immune-related hepatitis. Discontinuation of nivolumab resulted in the occurrence of left pelvic and inguinal pain. Following re-challenge with itraconazole, the patient has not reported any pain for 4 months.

**Conclusion:**

The findings of this case suggest that itraconazole is a potential effective treatment option for primary malignant melanoma of the vagina. Moreover, we identified potential itraconazole target genes, which could help elucidate the mechanism underlying this disease and potentially aid in the development of new therapeutic agents.

## Background

Primary malignant melanoma of the vagina is extremely rare and accounts for approximately 5% of vaginal cancers [1]. The prognosis of this condition is very poor compared to the prognosis of malignant melanoma arising from the skin because of the advanced clinical stage at presentation, and a recent report showed that the vagina being the primary site of melanoma is itself an independent prognostic factor [2]. The current standard treatment for unresectable or recurrent melanoma is immune checkpoint inhibitors, while RAF/MEK inhibitors are also used in symptomatic patients with *BRAF* mutation [3]. However, a second-line treatment regimen has not been established, and the development of novel treatments is warranted.

Itraconazole, a common oral anti-fungal agent, exerts antitumor activity by modulating the signal transduction pathways in cancer cells and cancer-associated fibroblasts, and by inhibiting angiogenesis [4]. Several clinical trials that investigated the repurposing of itraconazole as an anticancer agent against various types of cancers have yielded promising results [5–11]. Liang et al. suggested that itraconazole may inhibit melanoma growth by blocking Hedgehog, Wnt, and phosphatidylinositol 3-kinase-mammalian target of rapamycin (PI3K/mTOR) pathways, and showed that itraconazole prolonged survival in an in vivo xenograft mouse model of melanoma [12]. However, to our knowledge, the anti-cancer effect of itraconazole on melanoma has not been investigated in humans. Moreover, vaginal melanoma is a rare condition that is genetically and molecularly different from cutaneous melanoma [13], and it is not known whether itraconazole is effective against vaginal melanoma.

In the present report, we describe a case of metastatic, unresectable vaginal malignant melanoma treated with a 30-day course of oral itraconazole in a clinical window-of-opportunity trial, and an early clinical response was obtained. To the best of our knowledge, this is the first report of a case in which itraconazole was used to treat primary malignant melanoma of the vagina in a human patient, and in which its response and potential targets were evaluated by ^18^F-fluoro-deoxyglucose positron emission tomography (FDG/PET)–computed tomography (CT) and tissue cDNA microarray, respectively.

## Case presentation

A 64-year-old, multiparous Japanese woman was referred to our institution with a 1-year history of abnormal vaginal bleeding and a 2-month history of abnormal vaginal bleeding and left groin pain. Her medical and family history was unremarkable, and she was not taking any medication. A gynaecologic examination revealed a raised, dark-red necrotic lesion, 5 cm in diameter, located on the left lateral wall of the proximal vagina, and a non-pigmented thick tumour in the lower two-thirds of the posterior vagina. The cervix and fornix appeared normal. Her left groin was reddish and swollen, and enlarged inguinal lymph nodes were palpable with tenderness. Her left lower abdomen was oedematous. We performed a biopsy of the vaginal tumour, and arrived at a tentative diagnosis of unresectable vaginal cancer with metastasis to the inguinal lymph nodes.

The patient was informed about a window-of-opportunity clinical trial registered in the University Hospital Medical Information Network (UMIN 000018388), which included oral itraconazole therapy and tumour biopsies before and after itraconazole treatment. Informed consent was obtained from the patient for inclusion in the trial. Thus, on the first day of referral, she was started on 200 mg itraconazole orally twice a day, and we planned to continue this treatment regimen until the biopsy results were obtained and imaging studies were performed.

On day 1 of itraconazole treatment, her serum lactic dehydrogenase was slightly elevated (252 U/L) and squamous cell carcinoma antigen was normal (1.1 ng/mL). Her complete blood count and liver function were normal.

**Fig. 1:**
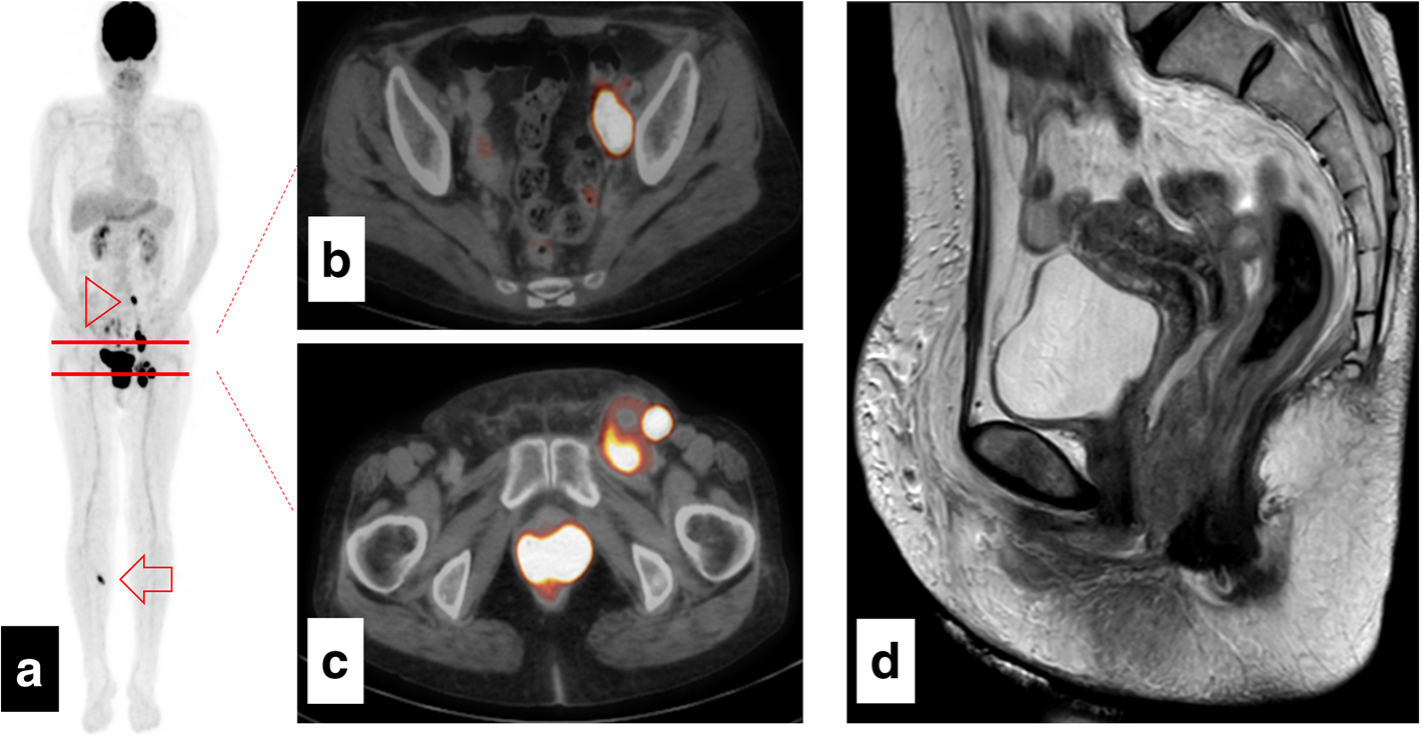
Images from ^18^F-fluoro-deoxyglucose positron emission tomography–computed tomography performed 5 days after the initiation of itraconazole (itraconazole) treatment (**a**, **b**, **c**). A magnetic resonance image acquired 12 days after the initiation of itraconazole treatment (**d**). **a** The arrowhead shows paraaortic lymph node metastases, and the arrow shows metastases to the right foot. **b** Pelvic lymph node metastases. **c** Left inguinal lymph node metastases. **d** Thickening of the lower two-thirds of the posterior vaginal wall

On day 6, FDG/PET–CT findings suggested a vaginal tumour and multiple metastases to the left inguinal, pelvic, and para-aortic lymph nodes, and right foot (Fig. 1a-c). The largest total diameter of the aggregated inguinal lymph nodes was 50 mm, and its maximum standard uptake value (SUVmax) was 19.8.

On day 7, she reported relief from her left inguinal pain. Physical examination revealed that her left inguinal nodes had shrunk and that the oedema had improved.

**Fig. 2:**
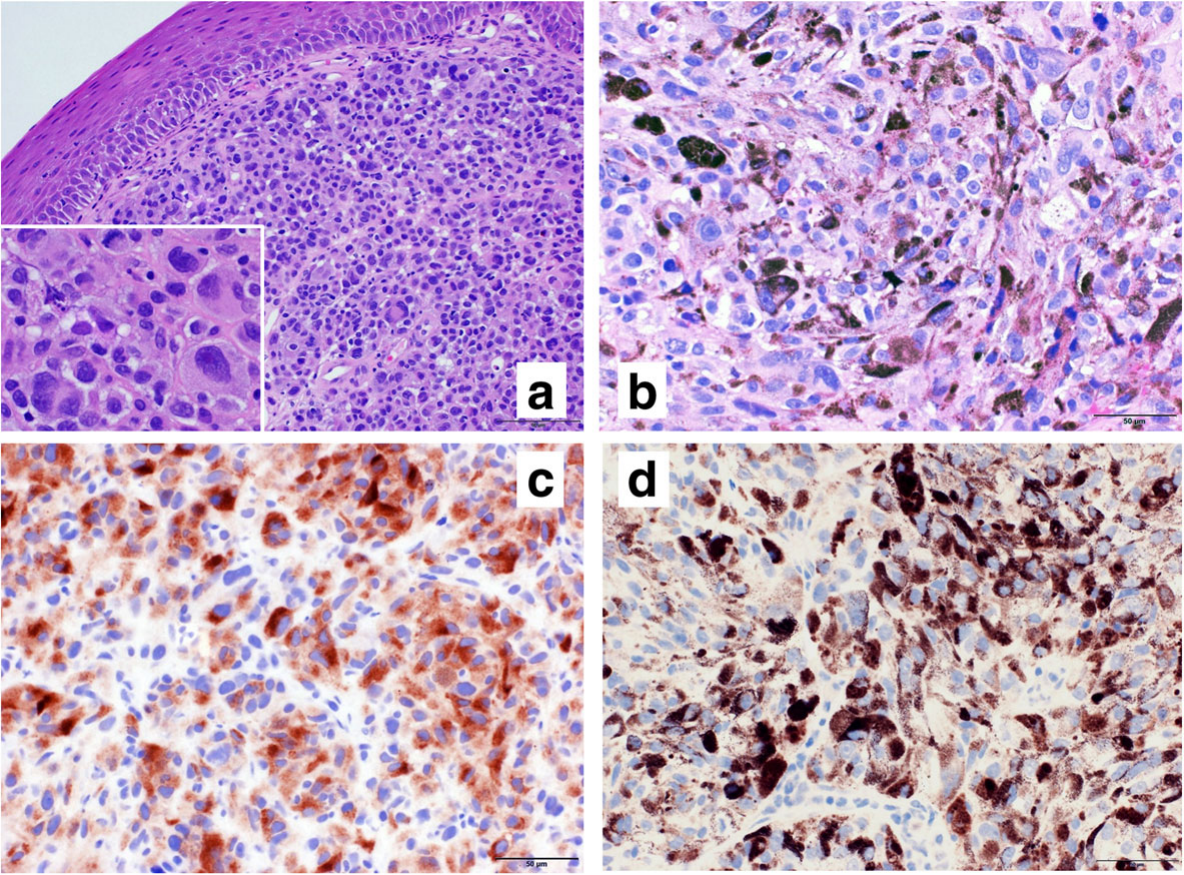
Histological findings of the vaginal malignant melanoma biopsied on the first day (before itraconazole treatment). **a** and **b** Haematoxylin and eosin staining. **a** Nested tumour cells can be observed under the normal squamous epithelium lining; the tumour cells show pleomorphism, multinuclei, bizarre nuclei, prominent nucleoli, and mitosis (inset). **b** Some tumour cells contain fine melanin granules. **c** Immunostaining with anti-Melan A antibody; most tumour cells shows strong cytoplasmic staining with anti-Melan A antibody. **d** Immunostaining with programmed death-ligand 1 (PD-L1) antibody (28–8) shows membranous and cytoplasmic staining in 20% of tumour cells

On day 9, microscopic evaluation of the biopsy sample confirmed the diagnosis of vaginal malignant melanoma, with positive immunohistochemical staining for HMB-45, S-100, and Melan A (Fig. 2).

On day 13, pelvic magnetic resonance imaging (MRI) revealed a thickened vaginal wall (Fig. 1b). We subsequently performed a second vaginal biopsy on the same day. Head MRI performed on day 14 revealed brain metastases. Each of the two brain tumours measured 1 cm in diameter, and the patient experienced no headache or neurological symptoms. On day 23, she underwent gamma-knife radiosurgery for brain metastases.

FDG/PET–CT performed on day 30 showed that the diameter of aggregated inguinal lymph nodes decreased to 38 mm, with an SUVmax of 14.6 (Table 1). Overall tumour response indicated stable disease according to the response evaluation criteria in solid tumours (RECIST) version 1.1 [14]. Genetic testing of the tumour yielded negative results for *BRAF* mutation, and immunohistochemical staining of programmed death-ligand 1 (PD-L1) on day 30 yielded a 20% positivity rate. Therefore, nivolumab was chosen for systemic treatment. Itraconazole was discontinued on day 30. A third vaginal biopsy was performed on day 30.

**Table 1:** ^18^F-fluoro-deoxyglucose positron emission tomography–computed tomography findings

Location		Day 6	Day 30
		SUVmax	SUVmax
Vagina		28	23
Lymph nodes (short axis)	Left pelvis	22.7	11.7
	Left inguinal	19.8	14.6
Right foot		9	7.4

The SUVmax levels of the primary vaginal tumour and metastatic tumours on day 6 and day 30 were compared. After treatment, SUVmax decreased in all the four areas. SUVmax, maximum standard uptake value

The biopsied specimens obtained on days 1, 13, and 30 were subjected to cDNA microarray analysis. Ribonucleic acid (RNA) was isolated from the specimens and subjected to cDNA microarray analysis using SurePrint G3 Human GE 8x60K v3 Microarray kit (Agilent Technologies, Tokyo, Japan). Altered gene expression was calculated as log2 (messenger RNA [mRNA] levels in itraconazole-treated tissues versus the mRNA level before itraconazole treatment). Genes down-regulated to < 1/32 of baseline levels after 13 days of itraconazole administration are listed in Table 2.

**Table 2:** The results of tissue cDNA microarray analysis

	Pre-treatment	Day 13		Day 30	
Gene symbol	Signal	Signal	Log2 ratio	Signal	Log2 ratio
*STATH*	14,257	50	−8.16	275	−5.7
*EEF1A2*	3329	16	−7.7	3847	0.21
*TTR*	164,093	790	−7.7	75,548	−1.12
*CDH2*	3933	37	−6.72	1716	−1.2
*FGF9*	5098	50	−6.66	792	−2.69
*WNT11*	1493	18	−6.38	77	−4.29
*MCHR1*	1183	17	−6.14	3568	1.59
*GNAO1*	1759	26	−6.07	1325	−0.41
*HS6ST2*	1019	15	−6.06	2396	1.23
*CA8*	1510	37	−5.34	4297	1.51
*PNPLA3*	11,614	344	−5.08	16,371	0.5
*AQP7*	5427	165	−5.04	20	−8.12

mRNA was isolated from tissues biopsied before treatment, and 13 and 30 days after the initiation of itraconazole. Genes down-regulated to < 1/32 of baseline levels after 13 days of itraconazole administration are listed in the table

During itraconazole treatment, the patient experienced no side effects and remained pain-free. Itraconazole also relieved her genital bleeding. In the 5 days between stopping itraconazole and starting nivolumab, her left inguinal nodes got enlarged and her left lower abdomen became oedematous, causing mild pain.

Initiation of nivolumab (3 mg/kg, repeated every 2 weeks) gradually improved her symptoms; however, the primary vaginal tumour increased in size after 10 weeks. After 12 weeks of nivolumab administration, the patient developed grade 3 immune-related hepatitis with elevated alanine transaminase (ALT; 194 U/l) but no bilirubin elevation. We discontinued nivolumab, and immediately administered 1 mg/kg of methylprednisolone. After 6 days, her liver enzyme levels promptly decreased to a grade 1 level. She developed left inguinal and left pelvic pain, which was treated with diclofenac rectal suppositories. FDG/PET–CT showed growth of the primary vaginal tumour and increased 18-FDG uptake in both the metastatic lymph nodes and the primary vaginal tumour. The patient was administered itraconazole, and her pain diminished the next day. During the 6-week steroid tapering, her ALT increased to 408 U/l, and 2 mg/kg of methylprednisolone was re-administered. After 3 months of itraconazole re-challenge, the primary vaginal tumour size gradually increased. However, a CT scan revealed that metastatic lymph nodes were stabilized, and no new metastatic lesions were found. Head MRI revealed remission of brain metastases. She has not reported any pain in the 4 months after the itraconazole re-challenge.

## Discussion and conclusions

This case report provides two new insights. Firstly, to our knowledge, this is the first study to report a clinical response achieved by itraconazole treatment of primary malignant melanoma, and to evaluate the response using PET–CT. Secondly, the biopsied specimens were analysed by cDNA microarray, providing information on changes in gene expression in response to itraconazole treatment.

Itraconazole treatment resulted in an early clinical response. The patient’s left inguinal lymph nodes were a useful target for visual and palpatory evaluation. Within a week of the first itraconazole challenge, her inguinal nodes were smaller on palpation, and her pain was relieved. She discontinued itraconazole after a window period designed in the clinical trial, and subsequently experienced left inguinal pain and oedema with tumour growth. The rapid progression after cessation of itraconazole appeared to be a ‘flare-up’ phenomenon, which has been reported after discontinuation of tyrosine kinase inhibitors [15, 16]. To monitor the response to itraconazole, we used FDG/PET–CT. Imaging of the aggregated inguinal lymph nodes showed a 24% reduction in tumour size and a 26% reduction in the SUVmax. Because itraconazole was found to be effective in this patient in a window-of-opportunity trial, it was used for treatment, at a lower cost and with minimal side effects, after discontinuation of nivolumab because of progressive disease and immune-related adverse events.

Malignant melanoma is highly vascular, and expression of vascular endothelial growth factor and its receptors are associated with poor prognosis [17, 18]. A recent prospective study investigating the repurposing of propranolol as an anti-angiogenesis agent showed improved disease-free survival among propranolol-treated patients with melanoma [19]. Itraconazole also possesses anti-angiogenetic activity. In 2007, Gupta et al. screened US Food and Drug Administration (FDA)-approved drugs, and identified itraconazole as a promising anti-angiogenic agent [20]. Therefore, itraconazole might inhibit the growth of melanoma cells by inhibiting angiogenesis, as well as melanoma-associated fibroblasts [21, 22].

A previous study investigated the anti-melanoma effect of itraconazole in vitro and in vivo in mice [12], and the results indicated that itraconazole suppresses the Hedgehog, Wnt, and PI3K/mTOR signalling pathways in melanoma cells. These signalling alterations were also demonstrated in cervical cancer CaSki cells treated with itraconazole [23]. In the present study, cDNA microarray analysis did not reveal any effects of itraconazole on Hedgehog or WNT/β-catenin pathways. Itraconazole interferes with multiple pathways in both cancer cells and the surrounding stromal cells, and a variety of effects on different types of cancer cells have been previously reported [4]. Itraconazole inhibited angiogenesis by inhibiting AKT (protein kinase B)/mTOR signalling in human umbilical vein endothelial cells, which was a result of both aberrant mitochondrial metabolism and blockage of cholesterol trafficking [24]. Using cell lines derived from vaginal melanoma and other malignancies, we found that itraconazole inhibited cholesterol transport in cancer cells (unpublished data).

In the present case, tissue cDNA microarray analysis revealed intriguing results. Transcriptions of four genes (*STATH, EEF1A2, TTR*, and *CDH2*) were down-regulated 100-fold. The *STATH* gene encodes statherin, *EEF1A2* encodes eukaryotic translation elongation factor 1 alpha 2, *TTR* encodes transthyretin, and *CDH2* encodes N-cadherin. These results differ markedly from those of Liang et al. in melanoma cells [12], and the downregulation of these four genes by itraconazole treatment has not been reported in previous preclinical and clinical reports [4]. This might be because our study was conducted in a human patient, and because the molecular interactions in vaginal melanoma might be different from those in cutaneous melanoma [13]. We found that itraconazole inhibited growth of vaginal melanoma cells in vitro using a cell line, but inhibition of Hedgehog, Wnt, or PI3K/mTOR pathways was not observed (unpublished data). Although the precise mechanism underlying the anti-tumour activity of itraconazole in this patient is unknown, the tissue cDNA microarray did reveal transcript alterations in melanoma cells as well as the surrounding microenvironment, providing clues to the mechanisms of itraconazole in human tissue.

Statherin has been reported to play a role in oral health, including putative protective activity against carcinogenesis [25]. Expression of statherin RNA or protein has not reported in any other organ in healthy humans [26], and, to the best of our knowledge, there have been no reports of statherin expression in any the other malignancy except oral cancer. Plitidepsin, which targets EFF1A2, has been studied in two clinical trials conducted in patients with melanoma [27, 28]. Duanmin et al. reported that EEF1A2 is highly expressed in pancreatic ductal adenocarcinoma, and is associated with lymph node metastasis [29]. It is not clear from the present case if these previous findings are applicable to vaginal malignant melanoma; further studies are needed to investigate this. A previous study on the use of serum biomarkers for the early detection of malignancy showed that transthyretin levels were elevated in melanoma as well as ovarian, endometrial, and lung cancer; however, the underlying mechanisms through which transthyretin is involved in these cancers remain unknown [30–33]. The transition from E-cadherin to N-cadherin is an essential step in the migration and invasion of cancer cells, including melanoma cells [34]. In vitro transfection of melanoma cells with small interfering RNA targeting the N-cadherin gene resulted in a decrease in matrix metalloproteinase-2 and -9 activity, and inhibited invasion [35].

In conclusion, itraconazole was effective in a patient with primary malignant melanoma of the vagina. The early response to itraconazole treatment was evaluated with FDG/PET–CT, and lymph node metastases were well-controlled. cDNA microarray analysis of the biopsied tissue of the vaginal tumour revealed down-regulation of genes that might be the targets of itraconazole. The interaction between itraconazole and these genes, and the effects of these interactions warrant further investigation in future studies.

## Abbreviations

AKT: Protein kinase B
ALT: Alanine transaminase
CT: Computed tomography
FDG/PET: 18F-fluoro-deoxyglucose positron emission tomography
MRI: Magnetic resonance imaging
mRNA: Messenger ribonucleic acid
mTOR: Mammalian target of rapamycin
PD-L1: Programmed death-ligand 1
PI3K: Phosphatidylinositol 3-kinase
SUVmax: Maximum standard uptake value
